# Relationship between maternal folic acid supplementation during pregnancy and risk of childhood asthma: Systematic review and dose-response meta-analysis

**DOI:** 10.3389/fped.2022.1000532

**Published:** 2022-11-17

**Authors:** Fushuang Yang, Jinpu Zhu, Zhongtian Wang, Lei Wang, Tianhui Tan, Liping Sun

**Affiliations:** ^1^ College of Chinese Medicine, Changchun University of Chinese Medicine, Changchun, China; ^2^ Center of Children's Clinic, The Affiliated Hospital to Changchun University of Chinese Medicine, Changchun, China

**Keywords:** folic acid, asthma, children, pregnancy, risks, dose-response meta-analysis

## Abstract

Growing evidence suggests that maternal folic acid supplementation during pregnancy may be associated with the risk of childhood asthma, but these findings remain controversial. Therefore, the purpose of this systematic review and meta-analysis was to assess the association between maternal folic acid supplementation during pregnancy and the risk of childhood asthma, and to determine the safe dose of folic acid supplementation during pregnancy based on a dose-response analysis to lower the risk of childhood asthma. The PubMed, Embase, Cochrane Library, and Web of Science databases were searched for relevant studies published before April 2022. The Newcastle-Ottawa Scale (NOS) was used to evaluate the quality of eligible studies, and a fixed-effect model was employed to calculate the odds ratio (OR) of asthma with 95% confidence intervals (CI). In addition, the generalized least-squares trend (GLST) was used to explore a nonlinear dose-response relationship. Stata 15.0 was used for the statistical analysis mentioned above. This systematic review included 18 studies (13 cohort studies, 5 case-control studies) with a total of 252,770 participants, 50,248 of whom were children with asthma. The meta-analysis showed that maternal folic acid supplementation during pregnancy was significantly associated with the risk of childhood asthma (OR = 1.07; 95% CI = 1.04–1.11). The subgroup analysis revealed a significant correlation between the risk of childhood asthma and the folic acid supplementation in the first Trimester (OR = 1.09; 95% CI = 1.05–1.12), the third Trimester (OR = 1.15; 95% CI = 1.04–1.26) and the whole pregnancy (OR = 1.13; 95% CI = 1.10–1.16). At the same time, the dose-response analysis showed a nonlinear relationship between maternal folic acid intake during pregnancy and the risk of childhood asthma. The risk of asthma in children significantly increased when maternal folic acid intake reached 581 μg/day. This meta-analysis showed that maternal folic acid supplementation during pregnancy increased the risk of asthma in children. Based on the results of the dose-response analysis, less than 580 μg folic acid per day is advised in order to effectively prevent birth defects without increasing the risk of childhood asthma.

**Systematic Review Registration:** https://www.crd.york.ac.uk/prospero/display_record.php?, identifier: CRD42022332140.

## Introduction

Asthma is the most prevalent chronic respiratory disease in children and adults, affecting approximately 334 million people worldwide. It is characterized by variable expiratory airflow restriction and recurrent symptoms, such as wheezing, shortness of breath, chest tightness, and cough (1, 2). Furthermore, asthma in children may represent 20% of the population (3). Asthma prevalence is steady or falling in many developed countries, but rising rapidly in developing countries where lifestyles are getting westernized (4, 5). Despite the widespread use of inhaled corticosteroids and the standardization of guidelines for asthma treatment, most children's asthma control remains suboptimal (6, 7). Although global asthma-related mortality continues to decrease (8), the high incidence of asthma in children leads to stunting (9), absenteeism (10), and increasing personal (11) and socioeconomic burdens (12). Therefore, identifying the risk factors for childhood asthma is important for primary prevention and early intervention of asthma (13, 14).

Asthma is caused by a complex gene-environment interaction. The occurrence of asthma is closely correlated with nutritional supplementation (15). Folic acid, an essential B vitamin, plays a key role in protein synthesis and cell division and growth, because it acts as a single-carbon donor in the synthesis of methionine, nucleotides, and pantothenic acid (16, 17). In addition, folic acid features in epigenetics by providing methyl groups for DNA methylation reactions (18). As a result, folic acid plays an irreplaceable role in people's health, especially in the early stages of life's growth and development (19). Several studies have reported that insufficient maternal folate levels during pregnancy may cause multiple birth defects in the fetus, such as neural tube defects (20), heart defects (21), and craniofacial malformations (22). Therefore, the World Health Organization recommends that all pregnant women should supplement and fortify folic acid in their diet to prevent birth defects (23). Some countries have even made folic acid fortification mandatory in recent years (24). However, supplementation combined with mandatory fortification has resulted in higher levels of folic acid and related metabolites in women of childbearing age (25). Recent studies have shown that excessive folic acid intake may harm the health of an offspring, such as impaired embryonic brain development (26), metabolic dysfunction (27), and allergic diseases (28).

In recent years, researchers have increasingly focused on the association between folic acid supplementation during pregnancy and the risk of childhood asthma, but their findings are inconsistent. Therefore, we conducted a comprehensive systematic review and meta-analysis based on available evidence to investigate (1) whether maternal folic acid supplementation during pregnancy is associated with the risk of childhood asthma; (2) whether there is a relationship between the occurrence of asthma in children and the daily intake of folic acid in mothers; (3) the relationship between folic acid supplementation and childhood asthma development at different stages (before conception, first trimester, second trimester, third trimester, whole trimester, and others); and (4) whether the association between maternal folic acid supplementation and the risk of childhood asthma varies with economic development levels of different countries.

## Methods

This meta-analysis was reported according to the PRISMA 2020 (The Preferred Reporting Items for Systematic Reviews and Meta-Analyses 2020) guideline and MOOSE (Meta-analysis of Observational Studies in Epidemiology) recommendations (29, 30). PROSPERO Registration ID: CRD42022332140.

## Search strategy

The PubMed, Embase, Cochrane Library, and Web of Science databases were retrieved for relevant studies published before April 12th, 2022. Both subject words (MeSH) and free words were searched. The search terms included “Folic Acid” [Mesh] and “Asthma” [Mesh]; the keywords were: “Folic Acid” OR “Vitamin M” OR “Vitamin B9” OR “B9, Vitamin” OR “Pteroylglutamic Acid” OR “Folvite” OR “Folacin” OR “Folate” in combination with “Asthma” OR “Asthmas”. The search strategy is shown in Supplementary Annex 1. In addition, the references of review articles were searched for potentially eligible studies.

## Selection criteria

This systematic review complied with the following inclusion and exclusion criteria to select eligible studies.

Inclusion criteria: original studies on the association between folic acid supplementation during pregnancy and the risk of childhood asthma; (2) cohort studies or case-control studies; (3) studies that provided risk evaluation of the association between childhood asthma and maternal daily folic acid intake or serum folate concentrations in women during their pregnancy; (4) studies published in English.

Studies with the following characteristics were excluded: (1) the sample size was too small (sample size <50); (2) there was no direct or indirect access to the odds ratio (OR) or relative risk (RR); (3) there were serious defects in the research data, and the literature was published in gray journals.

## Literature screening and data extraction

The retrieved studies were imported into EndNote X9. After removing duplicates, irrelevant studies were also deleted based on the titles and abstracts. Then the full texts of the remaining articles were downloaded and read to determine whether they could be finally included. The following data were extracted from all included studies: first author, date of publication, country and region, study design, source of participants, time of sampling, sample size, age, the period of folic acid supplementation, folic acid intake, statistical analysis, covariate adjustment, outcome measures, and other relevant characteristics. If several included studies reported ORs adjusted for different covariates, the ORs with the most adjusted covariates were extracted.

Literature screening and data extraction were independently carried out by two researchers (Y. F. S. and W. Z. T.) and cross-checked after completion. If there were any dissent, a third researcher (S. L. P.) was consulted to assist in the determination. If there was a lack of data, the researchers tried to contact the author to obtain it. If the information was inadequate, the researchers contacted the corresponding authors for more detailed data or other relevant information.

## Quality assessment

The Newcastle-Ottawa Scale (NOS) (31) was used to evaluate the quality of the included studies. The NOS scale comprises three domains with a total of eight items: four items for study subject selection, one for comparability between groups, and three for outcome measures. The total score ranges from 0 to 9 points. A score of 0–3, 4–6, and 7–9 is considered low quality, medium quality, and high quality, respectively.

## Statistical analysis

The meta-analysis was performed using Stata 15.0 (StataCorp, College Station, TX, United States), and the effect size was evaluated by OR with 95% confidence intervals (CI). The heterogeneity among the included studies was calculated by the *Q* test, and the heterogeneity index *I*^2^ was used to quantify the size of the heterogeneity. If *I*^2^ < 50%, a fixed-effects model was used for meta-analysis; if *I*^2 ^> 50%, a random-effects model was employed. Subgroup analyses and sensitivity analyses were conducted to explore potential sources of heterogeneity. A dose-response meta-analysis was also performed to explore the association between folic acid intake and the risk of childhood asthma. Restricted cubic spline models at four knots (10th, 35th, 65th, and 95th centiles) were established using the generalized least-squares trend (GLST). Furthermore, the cubic splines were used to model the nonlinear association between the daily dose of folic acid supplementation and childhood asthma. Accordingly, a dose-response nonlinear curve was plotted. A funnel plot was used to evaluate the publication bias, and Egger's and Begg's tests were also used to diagnose the publication bias. A *p* < 0.05 indicates the existence of publication bias. Under this circumstance, the impact of publication bias on the meta-analysis was evaluated using the trim-and-fill method. In this study, a *p* < 0.05 indicated that the difference was statistically significant.

## Results

### Literature search

**Figure 1:**
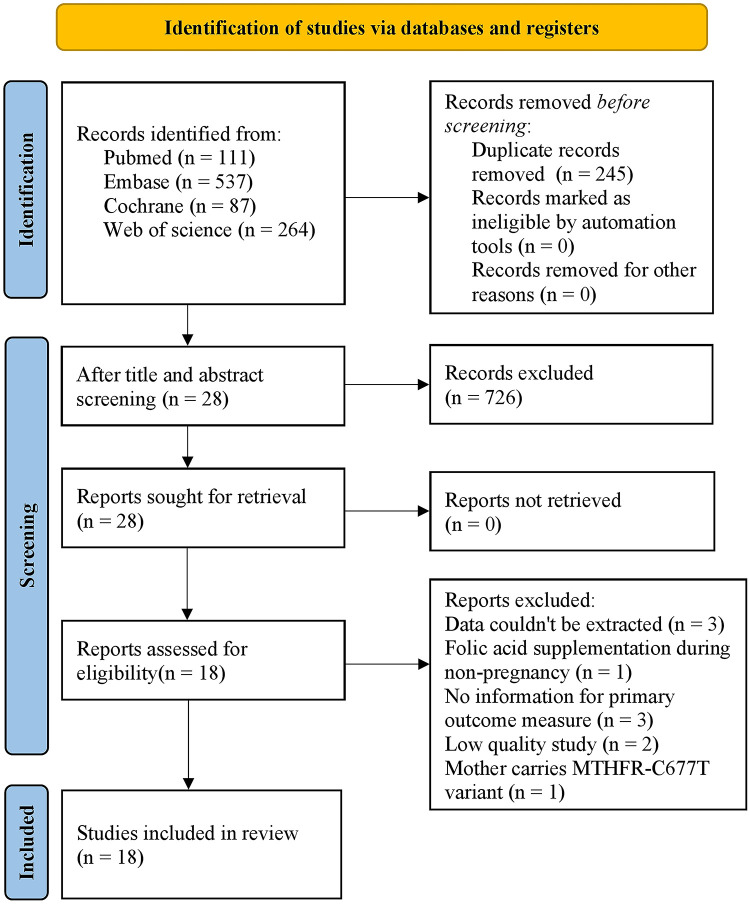
Flow diagram of literature selection.

Initially, 999 studies were retrieved from PubMed (*n* = 111), Embase (*n* = 537), Cochrane Library (*n* = 87) and Web of Science (*n* = 264). After the duplicates and irrelevant studies were removed based on the titles and abstracts, the full texts of the remaining 28 articles were downloaded and read to exclude ineligible studies according to the inclusion and exclusion criteria. Finally, 18 studies were included in this meta-analysis. The literature selection process is presented in Figure 1.

### Characteristics of the included studies and quality evaluation

Table 1 shows the characteristics of the included studies. A total of 18 (32–49) studies were eligible for our meta-analysis, including 13 cohort studies and five case-control studies. These studies were published between 2006 and 2022, involving 252,770 participants, including 50,248 children with asthma. Seven studies were conducted in Europe (37, 39, 42, 43, 45, 46, 48), five in North America (35, 36, 38, 40, 49), four in Asia (32–34, 44) and two in Australia (41, 47). The included studies adjusted for potential confounders, such as maternal age, race, parity, education level, smoking history, asthma history, infant sex, birth weight, mode of delivery, and feeding method. The NOS evaluation results are shown in Table 1. Overall, the scores for study quality ranged from 5 to 8. Twelve original studies (32, 33, 36–38, 40–42, 44–46, 49) were assessed as high quality, and six (34, 35, 39, 43, 47, 48) assessed as medium quality.

**Table 1:** Basic information for the included studies.

The first author (y)	Country	Study design	Sources of participants	Sampling time	No. of participants/cases	Age (years)	Folic acid intake	Statistical analysis	Adjustment for covariates	Outcome measure	Study quality
Chu S, 2022	China	Case-control study	Shanghai, China	2015.06–2016.01	1364/548	4–12	400–800 µg/D	Unconditional logistic regression models.	Maternal education levels, paternal education levels, family history of allergic diseases in any of his family members, child age, gender, birthweight, gestational age, delivered by caesarean section, newborn resuscitation, and feeding in the first 6 months.	Period of folic acid exposure supplementation	7
Miyashita C, 2021	Japan	Cohort study	Hokkaido, Japan	2008–2015.09	6651/732 (1 years of age); 6651/1087 (2 years of age); 6651/838 (4 years of age); 6651/466 (7 years of age)	1, 2, 4, and 7	—	Logistic regression analysis.	Maternal age, parity, delivery year, alcohol consumption during pregnancy, log10-transformed maternal cotinine level, maternal allergic history, paternal allergic history, annual household income, and sex of the child.	Folic acid exposure	8
Liu J, 2020	China	Case-control study	China	2000.12–2001.09	9090/109	4–6	400 µg/D	Logistic regression analysis.	Maternal age at child birth, education, occupation, and parity.	Folic acid exposure	6
Alfonso VH, 2018	United States	Case-control study	Los Angeles, United States	2006–2007	1176/465	3	—	Poisson regression models with robust error variance and a log link function.	Mother's race/ethnicity and nativity, mother's age at pregnancy, mother's education at the time of pregnancy, use of preconception vitamins, initiation of prenatal care, alcohol use during pregnancy, home environmental tobacco smoke during pregnancy, pre-pregnancy BMI, marital status, primary source of payment for prenatal care, parity, and birth outcome. Maternal history of atopy, duration of exclusive breastfeeding, child attendance to daycare or preschool, infection during pregnancy, and housing characteristics.	Folic acid exposure	6
Roy A, 2018	United States	Cohort study	Memphis, Tennessee, United States	2006–2011	849/174	3	10 ng/ml (Maternal plasma folate level)	Logistic regression analysis.	Maternal age at enrollment, self-reported race, education, prenatal smoking, asthma, pre-pregnancy body mass index, 2nd trimester vitamin D levels, parity, delivery route, and child sex and birth weight, breastfeeding.	Supplementary period	8
Parr CL, 2017	Norway	Cohort study	Norwegian birth registry and Norwegian Prescription Database	2014.04.01	39,846/1901	7	400 µg/D	Log binomial regression or multinomial logistic regressio.	Maternal age at delivery, parity, maternal education, prepregnancy body mass index, maternal smoking in pregnancy, and use of cod liver oil, other dietary supplements, and maternal energy intake in pregnancy.	Folic acid exposure supplement dosage	8
Veeranki SP, 2015	United States	Cohort study	Tennessee, United States	1996–2005	104,428/15,776	4.5–6	1000 µg/D	Multivariable logistic regression analysis.	Maternal characteristics included race, age at delivery, education, smoking during pregnancy, marital status, year of pregnancy, history of asthma, region of residence, and adequacy of prenatal care. Child characteristics included gender, birth weight, estimated gestational age and number of siblings.	Supplementary period	7
Zetstra-van der Woude PA, 2014	Netherlands	Case-control study	The pregnancy database IADB.nl, Netherlands	1994–2011	35,604/11,780	—	5000 µg/D	Logistic regression analysis.	Age of the mother, single or multiple pregnancy, maternal asthma medication, and paternal asthma medication. Dispension of iron supplements, antifolate medication, antidepressants, antihypertensives, antidiabetics, and benzodiazepines during pregnancy.	Folic acid exposure	6
Martinussen MP, 2012	United States	Cohort study	Massachusetts and Connecticut, United States	2003.09–2007.01	1499/223	6	Q1, 0 µg/D; Q2, <400 µg/D; Q3, 400–800 µg/D; Q4, >800 µg/D.	Logistic regression analysis. Variance (ANOVA) with Bonferroni and Scheffes Post Hoc tests.	Maternal parity, ethnicity and marital status, household income, maternal asthma, smoking during pregnancy, use of other vitamins (C, D and E), iron use, and calcium use in first trimester.	Supplementary period	8
Dunstan JA, 2012	Australia	Cohort study	Western Australia	—	628/59	1	Q1, <200 µg/D; Q2, 200–499 µg/D; Q3, >500 µg/D.	Associations between normally and lognormally distributed variables were evaluated in linear models. Logistic regression analysis.	Maternal age, maternal allergic disease, previous pregnancies, socioeconomic status, and education level. Infants' daycare attendance, infection history, postnatal dietary intervention, pet keeping, breast-feeding, and infant dietary patterns.	Folic acid exposure supplement dosage	8
Bekkers MB, 2012	Netherlands	Cohort study	Netherlands	2004–2005	3604/822, 3 years of age; 3484/653, 4 years of age; 3418/605, 5 years of age; 3389/496, 6 years of age; 3299/406, 7 years of age; 3237/419, 8 years of age.	1–8	—	Log binomial regression analyses.	Sex, birth weight, gestational age, number of older siblings, maternal education, maternal allergy, maternal body mass index before pregnancy, maternal smoking during pregnancy, maternal use of other vitamin supplements (A, C, D or E) than folic acid-only, prenatal and multivitamin or vitamin B complex supplements, maternal age at child birth, breast feeding duration, smoking in the home by anyone at 1 yr of age, type of day care at 1 yr of age and region.	Folic acid exposure	8
Kiefte-de Jong JC, 2012	Netherlands	Cohort study	Netherlands	2002.04–2006.1	8742/3409, 1 years of age; 8742/1923, 2 years of age; 8742/1311, 3, 4 years of age.	0–4	400–500 µg/D	Logistic GEE analyses.	Maternal age at pregnancy; maternal BMI at inclusion; maternal educational level; maternal ethnicity; infant's sex; infant's birth weight and gestational age at birth; any maternal smoking during pregnancy; any maternal alcohol consumption during pregnancy; duration of breastfeeding; any attendance of day care of the child in the first 24 mo of the infant's life; parental atopic constitution.	Period of folic acid exposure supplementation	6
											
Miyake Y, 2011	Japan	Cohort study	Neyagawa City, Japan	2001.11–2003.03	763/169	16–24 months	Q1, 206.8 µg/D; Q2, 255.1 µg/D; Q3, 291.2 µg/D; Q4, 370.6 µg/D.	Logistic regression analysis; Multiple logistic regression analysis.	Adjustment for maternal age, gestation at baseline, residential municipality at baseline, family income, maternal and paternal education, maternal and paternal history of asthma, atopic eczema, and allergic rhinitis, changes in maternal diet in the previous 1 month, season when data at baseline were collected, maternal smoking during pregnancy, baby's older siblings, baby's sex, baby's birth weight, household smoking in same room as infant, breastfeeding duration, age at which solid foods were introduced, age of infant at the third survey, and maternal intake of docosahexaenoic acid, n-6 polyunsaturated fatty acids, vitamin D, calcium, vitamin E, and *β*-carotene during pregnancy.	Folic acid exposure supplement dosage	7
Magdelijns FJ, 2011	Netherlands	Cohort study	the KOALA study, Netherlands	2002.01	2640/130	6–7	—	Univariable and multivariable logisticregression analysis.	Recruitment group, maternal antibiotic, smoking and alcohol use during pregnancy, mode and place of delivery, birth weight, gender, treatment with antibiotics during the first 6 months of life, exposure to environmental tobacco smoke and domestic animals, breastfeeding, maternal education level, family history of atopy, siblings, day care attendance, and multivitamin or other supplement use during pregnancy.	Period of folic acid exposure supplementation	8
Håberg SE, 2011	Norway	Case-control study	the MoBa study, Norway	2002.07–2004.06	1962/507	3	Q1, <5.54 nmol/l; Q2, 5.54–7.68 nmol/l; Q3, 7.68–10.6 nmol/l; Q4, 10.6–17.84 nmol/l; Q5, >17.84 nmol/l. (Maternal plasma folate level)	Univariate and multivariate logistic regression analysis.	Maternal atopy, maternal educational level, parity, maternal prepregnancy body mass index (BMI) calculated from height and prepregnancy weight, maternal smoking in pregnancy, maternal smoking when the child was three years, and the child's use of vitamin supplements or cod liver oil at three years of age.	Folic acid exposure	8
Whitrow MJ, 2009	Australia	Cohort study	Adelaide, Australia	1998–2005	557/57, 3.5 years of age; 557/50, 5.5 years of age.	3.5, 5.5	400 µg/D	Poisson regression model.	—	Supplementary period	5
Håberg SE, 2009	Norway	Cohort study	the MoBa study, Norway	2000.01–2005.06	32,077/12,656	6–18 months	400 µg/D	Generalized linear model.	Other supplements in pregnancy, sex, birth weight, month of birth, and maternal atopy, maternal educational level, parity, maternal smoking in pregnancy, type of day care, parental smoking in first 3 months after birth, breast feeding at 6 months, and exposure to vitamin supplements or cod liver oil at 6 months of age.	Supplementary period	6
Litonjua AA, 2006	United States	Cohort study	Boston, United States	1999.04–2002.07	1290/376	2	400 µg/D	Bivariate logistic regression models.	Birth weight, neonate sex, maternal age, maternal prepregnancy body mass index, breastfeeding duration, the number of children <12 y old in the home, postnatal passive smoke exposure, family income, and maternal and paternal asthma.	Folic acid exposure	8

### Relationship between folic acid supplementation during pregnancy and the risk of childhood asthma

Of the 18 included studies, 13 studies (32–35, 37, 39, 41–46, 49) reported an association between maternal folic acid supplementation during pregnancy and the risk of asthma in children. A fixed-effect model (*I*^2^ = 21.8%) was used to pool effect sizes. The OR of maternal folic acid supplementation was 1.07 (95% CI = 1.04–1.11; *P* = 0.128), indicating that maternal folic acid supplementation during pregnancy was significantly associated with the risk of childhood asthma. Sensitivity analyses showed that deleting any single study had no significant effect on the overall OR.

**Figure 2:**
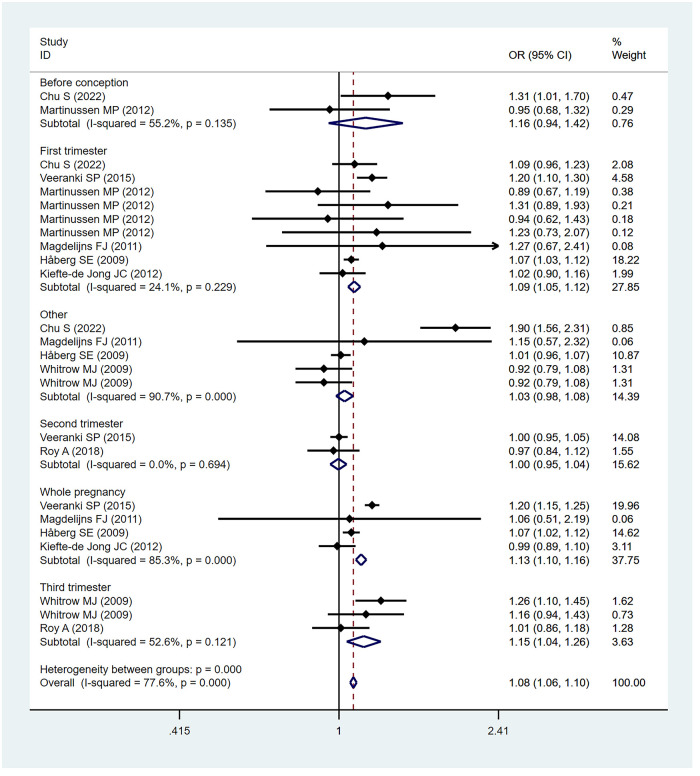
Subgroup analysis based on folic acid supplementation in different periods of pregnancy.

Eight studies (32, 36, 38, 40, 43, 45, 47, 48) found a link between maternal folic acid supplementation at different times and the risk of childhood asthma. A subgroup analysis was performed based on the folic acid supplementation at different stages of pregnancy. The subgroup analysis found a significant association between the risk of childhood asthma and the folic acid supplementation in the first trimester (OR = 1.09; 95% CI = 1.05–1.12), the third trimester (OR = 1.15; 95% CI = 1.04–1.26), and the whole pregnancy (OR = 1.13; 95% CI = 1.10–1.16). Since few studies focus on prefecundation and the second trimester, we failed to explore the association between folic acid supplementation and childhood asthma risk during the two periods based on available evidence. The subgroup analysis based on folic acid supplementation in different periods of pregnancy is shown in Figure 2.

Another subgroup analysis was conducted according to the economic development level of different countries. Eleven studies (33, 35, 37, 39, 41–46, 49) were included in the analysis of high-income economies (OR = 1.05; 95% CI = 1.01–1.09), and two studies (32, 34) were included in the analysis of middle-income economies (OR = 1.26; 95% CI = 1.13–1.41). However, no literature was available for the analysis of low-income economies. According to this subgroup analysis, folic acid supplementation during pregnancy increased the risk of asthma in children regardless of the economic development levels.

### Dose-response analysis

**Figure 3:**
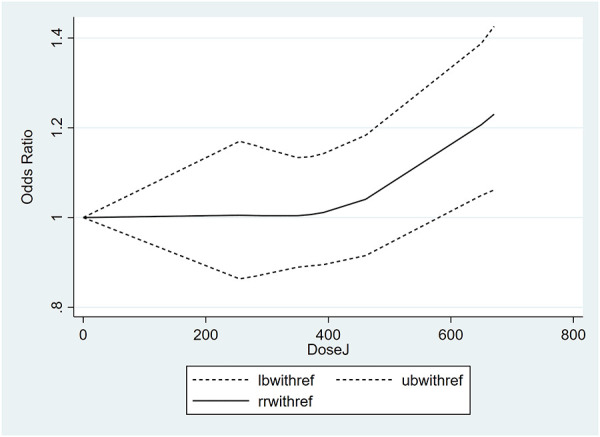
Dose-response analysis of daily maternal folic acid intake and risk of childhood asthma.

Studies with relevant data were selected for a dose-response analysis (37, 41, 44). The results of the dose-response analysis showed a nonlinear relationship between maternal folate intake during pregnancy and childhood asthma risk. Maternal folate intake of less than 581 µg/day had no association with childhood asthma risk, whereas the intake of 581 µg/day or more significantly increased the risk of childhood asthma. The dose-response nonlinear curve is shown in Figure 3.

### Publication bias

**Figure 4:**
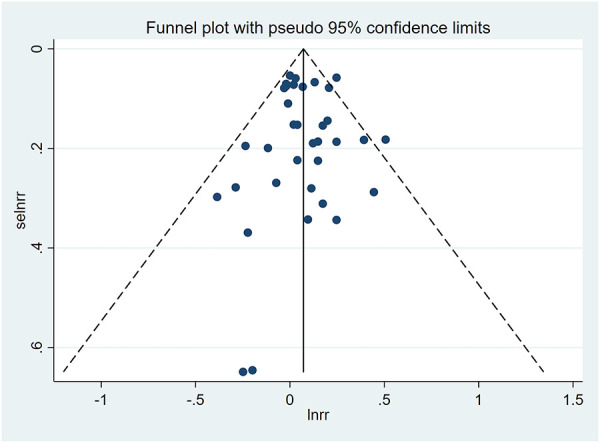
Funnel diagram.

**Figure 5:**
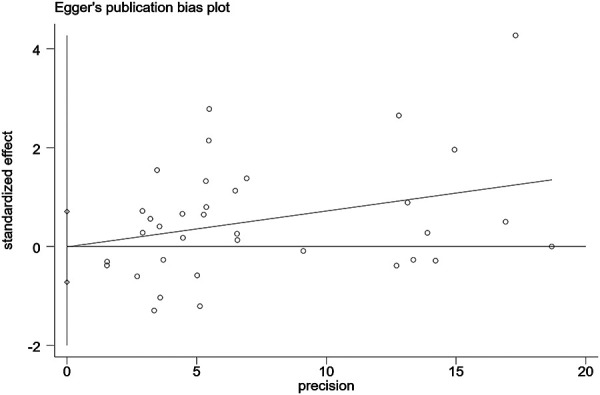
Egger's test.

A funnel plot was plotted to test the publication bias. The results showed that the left and right distributions were symmetrical, as shown in Figure 4. Neither Egger's test (*P* = 0.982) nor Begg's test funnel plot revealed publication bias. The results of Egger's test are shown in Figure 5.

## Discussion

The results of the meta-analysis suggested that maternal folic acid supplementation during pregnancy was associated with the risk of childhood asthma. According to subgroup analyses, the effects of folic acid supplementation were found to be significant in the first trimester, the third trimester, and the whole pregnancy. In addition, folic acid supplementation during pregnancy increased the risk of childhood asthma regardless of the economic development levels of different countries. The dose-response analysis showed a nonlinear relationship between maternal folic acid intake during pregnancy and the risk of childhood asthma. The maternal folic acid intake of less than 581 µg per day is not correlated with the risk of childhood asthma. However, the risk of childhood asthma significantly increases when the intake reaches 581 µg or more per day.

Litonjua AA. et al. (49) were the first to study the association between maternal folic acid supplementation during pregnancy and childhood asthma. Since then, researchers have been increasingly interested in this topic, but their findings are inconsistent and conflicting. This association was summarized in four previous meta-analyses. Krista et al. (50) conducted a meta-analysis of 5 studies, which showed that folic acid supplementation had no association with an increased risk of childhood asthma between preconception and the first trimester (RR = 1.01, 95% CI = 0.78–1.30); in addition, a meta-analysis of 5 studies by Yang L et al. (51) reported the same results (OR = 1.06, 95% CI = 0.99–1.14). However, a meta-analysis by Wang T et al. (52) suggested that maternal folic acid supplementation in early pregnancy may increase the risk of asthma in young children (RR = 1.06, 95% CI = 1.02–1.09); Li W et al. (53) also reported that maternal folic acid exposure during pregnancy was significantly associated with infant asthma risk (RR = 1.11; 95% CI = 1.06–1.17). Since the last meta-analysis was published, six more related studies with inconsistent results have emerged, allowing for more robust estimation and quantification. Given the controversy over the association between maternal folic acid supplementation during pregnancy and the risk of childhood asthma, we included new and updated studies for further meta-analysis to thoroughly investigate this relationship and clarify the dose-response association between maternal folic acid intake and childhood asthma.

The relationship between folic acid supplementation during pregnancy and childhood asthma is under exploration. Current evidence suggests that DNA methylation plays a key role in this process (54). DNA methylation is catalyzed by the enzymes that transfer methyl groups (methyl-transferases) from the methyl agent S-adenosylmethionine to cytosine. It is an epigenetic modification that is essential for normal genome regulation and development (55). Folate is a key source of the one-carbon group used to methylate DNA. Hollingsworth JW et al. (56) found that high-methyl donor diets may increase the risk of allergic airway disease in children through DNA methylation and transcription of abnormal genes. Studies also found that Runx3 mRNA (Runt-related transcription factor 3, a gene known to negatively regulate allergic airway disease) and protein levels were suppressed in offspring exposed to a hypermethylated (overmethylated) diet *in utero*. İscan B et al. (57) found that maternal folic acid supplementation during pregnancy affected offspring's airway remodeling and increased allergic reactions caused by offspring's ovalbumin excitation; additionally, the intensity of the response increased with the duration of supplementation and the accumulative dose. Despite an increasing number of related studies, we recognize that the mechanisms of folic acid inducing asthma in children remain unknown.

WHO and most countries recommend that pregnant women should maintain a healthy diet and take folic acid supplementation of 400 micrograms/day to prevent birth defects (58). According to this dose-response analysis, the risk of asthma in children significantly increased when the maternal folate intake reached 581 μg/day. A study with similar results suggested that maternal folic acid supplementation at a high dose during pregnancy was associated with an increased risk of asthma in infants, while a relatively low dose reduced the risk of asthma in infants (51). This reveals that although folic acid can effectively prevent birth defects, the adverse effects of high-dose supplementation on the health of children cannot be ignored. Therefore, how to safely supplement folic acid during pregnancy needs to be explored and verified by relevant research. In the subgroup analysis, we found that folic acid supplementation was significantly associated with the risk of asthma in children in the first trimester, the third trimester, and the whole pregnancy. Recommendations vary from country to country, but most advise folic acid supplementation from the first trimester (4 to 12 weeks) to the end of the second trimester (8 to 12 weeks) (58). Given that the neural tube closes around the 28th day of the embryo, the critical period for folic acid supplementation is in the first and second trimesters (59). The need for folic acid supplementation at other stages of pregnancy and its impact on the risk of childhood asthma requires further studies to confirm.

There are several advantages to our study. First, our analysis included 18 relevant studies, including those published in 2022. It is more statistically convincing than previous studies due to newer and larger sample sizes. Second, a subgroup analysis was conducted according to the different folic acid supplementation periods to explore the effect of folic acid supplementation at different periods on the risk of childhood asthma. Third, we made full use of the dose data of the included studies to conduct a dose-response analysis, which quantitatively revealed the relationship between folic acid intake during pregnancy and the risk of childhood asthma based on a qualitative summary. A dose-response curve was drawn, which may help develop strategies for safe folic acid supplementation during pregnancy. Finally, there is no publication bias in our analysis. However, some limitations of the present study should also be taken into account. First, all included studies adjusted for multiple confounding factors, but these factors were inconsistent and the effects of other confounding factors could not be excluded. Second, it is difficult to accurately calculate the dose of folic acid that pregnant women consume from both natural food and synthetics (vitamin supplements or prenatal fortification supplements). Third, the age of study participants varied widely from less than one year old to twelve years old, which might lead to a bias in the study results. Finally, only three studies were included in the dose-response analysis; therefore further dose-response studies are required for further validation.

## Conclusion

Maternal folic acid supplementation during pregnancy increases the risk of childhood asthma. At the same time, dose-response analysis testified a nonlinear relationship between folic acid intake during pregnancy and the risk of childhood asthma. When the maternal folate intake is ≥581 μg/day, the risk of asthma in children significantly increases. Although folic acid supplementation during pregnancy can prevent birth defects, its adverse effects on the health of offspring cannot be ignored. Therefore, we recommend that the daily dose of folic acid supplementation for pregnant women should be less than 580 μg, which can effectively prevent birth defects without increasing the risk of asthma in children.

## Data Availability

The original contributions presented in the study are included in the article/**Supplementary Material**, further inquiries can be directed to the corresponding author/s.

## References

[1] Aaron SD Boulet LP Reddel HK Gershon AS. Underdiagnosis and overdiagnosis of asthma. Am J Respir Crit Care Med. (2018) 198:1012–20. 10.1164/rccm.201804-0682CI 29756989

[2] Vos T Flaxman AD Naghavi M Lozano R Michaud C Ezzati M Years lived with disability (YLDs) for 1160 sequelae of 289 diseases and injuries 1990–2010: a systematic analysis for the global burden of disease study 2010. Lancet. (2012) 380:2163–96. 10.1016/s0140-6736(12)61729-2 23245607 PMC6350784

[3] Chetta A Calzetta L. Bronchial asthma: an update. Minerva Med. (2022) 113:1–3. 10.23736/s0026-4806.21.07958-1 34913639

[4] Papi A Brightling C Pedersen SE Reddel HK. Asthma. Lancet. (2018) 391:783–800. 10.1016/S0140-6736(17)33311-1 29273246

[5] Stern J Pier J Litonjua AA. Asthma epidemiology and risk factors. Semin Immunopathol. (2020) 42:5–15. 10.1007/s00281-020-00785-1 32020334

[6] Fitzpatrick AM Bacharier LB Jackson DJ Szefler SJ Beigelman A Cabana M Heterogeneity of mild to moderate persistent asthma in children: confirmation by latent class analysis and association with 1-year outcomes. J Allergy Clin Immunol Pract. (2020) 8:2617–2627.e4. 10.1016/j.jaip.2020.02.032 32156610 PMC7483393

[7] Sullivan PW Ghushchyan V Kavati A Navaratnam P Friedman HS Ortiz B. Trends in asthma control, treatment, health care utilization, and expenditures among children in the United States by place of residence: 2003–2014. J Allergy Clin Immunol Pract. (2019) 7:1835–42.e2. 10.1016/j.jaip.2019.01.055 30772478

[8] Beasley R Semprini A Mitchell EA. Risk factors for asthma: is prevention possible? Lancet. (2015) 386:1075–85. 10.1016/s0140-6736(15)00156-7 26382999

[9] Loke YK Blanco P Thavarajah M Wilson AM. Impact of inhaled corticosteroids on growth in children with asthma: systematic review and meta-analysis. PLoS ONE. (2015) 10:e0133428. 10.1371/journal.pone.0133428 26191797 PMC4507851

[10] Pijnenburg MW Fleming L. Advances in understanding and reducing the burden of severe asthma in children. Lancet Respir Med. (2020) 8:1032–44. 10.1016/s2213-2600(20)30399-4 32910897

[11] Bui AL Dieleman JL Hamavid H Birger M Chapin A Duber HC Spending on children's personal health care in the United States, 1996–2013. JAMA Pediatr. (2017) 171:181–9. 10.1001/jamapediatrics.2016.4086 28027344 PMC5546095

[12] Dieleman JL Cao J Chapin A Chen C Li Z Liu A US Health care spending by payer and health condition, 1996–2016. JAMA. (2020) 323:863–84. 10.1001/jama.2020.0734 32125402 PMC7054840

[13] von Mutius E Smits HH. Primary prevention of asthma: from risk and protective factors to targeted strategies for prevention. Lancet. (2020) 396:854–66. 10.1016/s0140-6736(20)31861-4 32910907

[14] Murray CS Jackson DJ Teague WG. Prevention and outpatient treatment of asthma exacerbations in children. J Allergy Clin Immunol Pract. (2021) 9:2567–76. 10.1016/j.jaip.2021.03.035 34246433

[15] Alwarith J Kahleova H Crosby L Brooks A Brandon L Levin SM The role of nutrition in asthma prevention and treatment. Nutr Rev. (2020) 78:928–38. 10.1093/nutrit/nuaa005 32167552 PMC7550896

[16] Mc Auley MT Mooney KM Salcedo-Sora JE. Computational modelling folate metabolism and DNA methylation: implications for understanding health and ageing. Brief Bioinform. (2018) 19:303–17. 10.1093/bib/bbw116 28007697

[17] Froese DS Fowler B Baumgartner MR. Vitamin B(12), folate, and the methionine remethylation cycle-biochemistry, pathways, and regulation. J Inherit Metab Dis. (2019) 42:673–85. 10.1002/jimd.12009 30693532

[18] Ly A Hoyt L Crowell J Kim YI. Folate and DNA methylation. Antioxid Redox Signal. (2012) 17:302–26. 10.1089/ars.2012.4554 22332737

[19] Wang G Hu FB Mistry KB Zhang C Ren F Huo Y Association between maternal prepregnancy body mass index and plasma folate concentrations with child metabolic health. JAMA Pediatr. (2016) 170:e160845. 10.1001/jamapediatrics.2016.0845 27295011 PMC5147730

[20] Clarke R Bennett D. Folate and prevention of neural tube defects. Br Med J. (2014) 349:g4810. 10.1136/bmj.g4810 25073785

[21] Czeizel AE Dudás I Vereczkey A Bánhidy F. Folate deficiency and folic acid supplementation: the prevention of neural-tube defects and congenital heart defects. Nutrients. (2013) 5:4760–75. 10.3390/nu5114760 24284617 PMC3847759

[22] Maldonado E Martínez-Sanz E Partearroyo T Varela-Moreiras G Pérez-Miguelsanz J. Maternal folic acid deficiency is associated to developing nasal and palate malformations in mice. Nutrients. (2021) 13:251. 10.3390/nu13010251 33467180 PMC7830789

[23] WHO Guidelines Approved by the Guidelines Review Committee, in Guideline: Optimal Serum and Red Blood Cell Folate Concentrations in Women of Reproductive Age for Prevention of Neural Tube Defects. (2015). Geneva: World Health Organization Copyright © World Health Organization (2015). 25996016

[24] Wald NJ Hoffbrand AV. Mandatory UK folic acid fortification. Lancet. (2021) 398:1961–2. 10.1016/s0140-6736(21)02447-8 34838169

[25] Murray LK Smith MJ Jadavji NM. Maternal oversupplementation with folic acid and its impact on neurodevelopment of offspring. Nutr Rev. (2018) 76:708–21. 10.1093/nutrit/nuy025 30010929

[26] Shulpekova Y Nechaev V Kardasheva S Sedova A Kurbatova A Bueverova E The concept of folic acid in health and disease. Molecules. (2021) 26:3731. 10.3390/molecules26123731 34207319 PMC8235569

[27] Tojal A Neves C Veiga H Ferreira S Rodrigues I Martel F Perigestational high folic acid: impact on offspring's peripheral metabolic response. Food Funct. (2019) 10:7216–26. 10.1039/c9fo01807g 31612177

[28] Molloy J Collier F Saffery R Allen KJ Koplin JJ Louise Ponsonby A Folate levels in pregnancy and offspring food allergy and eczema. Pediatr Allergy Immunol. (2020) 31:38–46. 10.1111/pai.13128 31566807

[29] Page MJ Moher D Bossuyt PM Boutron I Hoffmann TC Mulrow CD PRISMA 2020 Explanation and elaboration: updated guidance and exemplars for reporting systematic reviews. Br Med J. (2021) 372:n160. 10.1136/bmj.n160 33781993 PMC8005925

[30] Stroup DF Berlin JA Morton SC Olkin I Williamson GD Rennie D Meta-analysis of observational studies in epidemiology: a proposal for reporting. Meta-analysis of observational studies in epidemiology (MOOSE) group. JAMA. (2000) 283:2008–12. 10.1001/jama.283.15.2008 10789670

[31] Stang A. Critical evaluation of the Newcastle-Ottawa scale for the assessment of the quality of nonrandomized studies in meta-analyses. Eur J Epidemiol. (2010) 25:603–5. 10.1007/s10654-010-9491-z 20652370

[32] Chu S Zhang J. Periconceptional folic acid supplementation is a risk factor for childhood asthma: a case-control study. BMC Pregnancy Childbirth. (2022) 22:220. 10.1186/s12884-022-04567-5 35303823 PMC8933875

[33] Miyashita C Araki A Miura R Ait Bamai Y Kobayashi S Itoh S Prevalence of childhood wheeze and modified DNA methylation at 7 years of age according to maternal folate levels during pregnancy in the Hokkaido study. Pediatr Allergy Immunol. (2021) 32:514–23. 10.1111/pai.13425 33274524

[34] Liu J Li Z Ye R Liu J Ren A. Periconceptional folic acid supplementation and risk of parent-reported asthma in children at 4–6 years of age. ERJ Open Res. (2020) 6:00250–2019. 10.1183/23120541.00250-2019 32280668 PMC7132036

[35] Alfonso VH Bandoli G von Ehrenstein O Ritz B. Early folic acid supplement initiation and risk of adverse early childhood respiratory health: a population-based study. Matern Child Health J. (2018) 22:111–9. 10.1007/s10995-017-2360-6 28887720

[36] Roy A Kocak M Hartman TJ Vereen S Adgent M Piyathilake C Association of prenatal folate status with early childhood wheeze and atopic dermatitis. Pediatr Allergy Immunol. (2018) 29:144–50. 10.1111/pai.12834 29168294 PMC6087709

[37] Parr CL Magnus MC Karlstad Ø Haugen M Refsum H Ueland PM Maternal folate intake during pregnancy and childhood asthma in a population-based cohort. Am J Respir Crit Care Med. (2017) 195:221–8. 10.1164/rccm.201604-0788OC 27518161 PMC5394786

[38] Veeranki SP Gebretsadik T Mitchel EF Tylavsky FA Hartert TV Cooper WO Maternal folic acid supplementation during pregnancy and early childhood asthma. Epidemiology. (2015) 26:934–41. 10.1097/ede.0000000000000380 26360371 PMC4900760

[39] Zetstra-van der Woude PA De Walle HE Hoek A Bos HJ Boezen HM Koppelman GH Maternal high-dose folic acid during pregnancy and asthma medication in the offspring. Pharmacoepidemiol Drug Saf. (2014) 23:1059–65. 10.1002/pds.3652 24930442

[40] Martinussen MP Risnes KR Jacobsen GW Bracken MB. Folic acid supplementation in early pregnancy and asthma in children aged 6 years. Am J Obstet Gynecol. (2012) 206:72.e1–7. 10.1016/j.ajog.2011.07.033 PMC3246127 21982024

[41] Dunstan JA West C McCarthy S Metcalfe J Meldrum S Oddy WH The relationship between maternal folate status in pregnancy, cord blood folate levels, and allergic outcomes in early childhood. Allergy. (2012) 67:50–7. 10.1111/j.1398-9995.2011.02714.x 21923665

[42] Bekkers MB Elstgeest LE Scholtens S Haveman-Nies A de Jongste JC Kerkhof M Maternal use of folic acid supplements during pregnancy, and childhood respiratory health and atopy. Eur Respir J. (2012) 39:1468–74. 10.1183/09031936.00094511 22034647

[43] Kiefte-de Jong JC Timmermans S Jaddoe VW Hofman A Tiemeier H Steegers EA High circulating folate and vitamin B-12 concentrations in women during pregnancy are associated with increased prevalence of atopic dermatitis in their offspring. J Nutr. (2012) 142:731–8. 10.3945/jn.111.154948 22399526

[44] Miyake Y Sasaki S Tanaka K Hirota Y. Maternal B vitamin intake during pregnancy and wheeze and eczema in Japanese infants aged 16–24 months: the Osaka maternal and child health study. Pediatr Allergy Immunol. (2011) 22:69–74. 10.1111/j.1399-3038.2010.01081.x 20561231

[45] Magdelijns FJ Mommers M Penders J Smits L Thijs C. Folic acid use in pregnancy and the development of atopy, asthma, and lung function in childhood. Pediatrics. (2011) 128:e135–44. 10.1542/peds.2010-1690 21690114

[46] Håberg SE London SJ Nafstad P Nilsen RM Ueland PM Vollset SE Maternal folate levels in pregnancy and asthma in children at age 3 years. J Allergy Clin Immunol. (2011) 127:262–264.e1. 10.1016/j.jaci.2010.10.004 21094522 PMC3108064

[47] Whitrow MJ Moore VM Rumbold AR Davies MJ. Effect of supplemental folic acid in pregnancy on childhood asthma: a prospective birth cohort study. Am J Epidemiol. (2009) 170:1486–93. 10.1093/aje/kwp315 19880541

[48] Håberg SE London SJ Stigum H Nafstad P Nystad W. Folic acid supplements in pregnancy and early childhood respiratory health. Arch Dis Child. (2009) 94:180–4. 10.1136/adc.2008.142448 19052032 PMC3612898

[49] Litonjua AA Rifas-Shiman SL Ly NP Tantisira KG Rich-Edwards JW Camargo CA Jr Maternal antioxidant intake in pregnancy and wheezing illnesses in children at 2 year of age. Am J Clin Nutr. (2006) 84:903–11. 10.1093/ajcn/84.4.903 17023719 PMC1994925

[50] Crider KS Cordero AM Qi YP Mulinare J Dowling NF Berry RJ. Prenatal folic acid and risk of asthma in children: a systematic review and meta-analysis. Am J Clin Nutr. (2013) 98:1272–81. 10.3945/ajcn.113.065623 24004895 PMC5369603

[51] Yang L Jiang L Bi M Jia X Wang Y He C High dose of maternal folic acid supplementation is associated to infant asthma. Food Chem Toxicol. (2015) 75:88–93. 10.1016/j.fct.2014.11.006 25449200

[52] Wang T Zhang HP Zhang X Liang ZA Ji YL Wang G. Is folate Status a risk factor for asthma or other allergic diseases? Allergy Asthma Immunol Res. (2015) 7:538–46. 10.4168/aair.2015.7.6.538 26333700 PMC4605926

[53] Li W Xu B Cao Y Shao Y Wu W Zhou J Association of maternal folate intake during pregnancy with infant asthma risk. Sci Rep. (2019) 9:8347. 10.1038/s41598-019-44794-z 31171831 PMC6554315

[54] Crider KS Yang TP Berry RJ Bailey LB. Folate and DNA methylation: a review of molecular mechanisms and the evidence for folate's role. Adv Nutr. (2012) 3:21–38. 10.3945/an.111.000992 22332098 PMC3262611

[55] Han Y-Y Celedón J Medicine CC. Maternal folate intake during pregnancy and childhood asthma. Pittsburgh, Pennsylvania: American Thoracic Society (2017). 155–6. 10.1164/rccm.201608-1713ED PMC5394792 28084816

[56] Hollingsworth JW Maruoka S Boon K Garantziotis S Li Z Tomfohr J In utero supplementation with methyl donors enhances allergic airway disease in mice. J Clin Invest. (2008) 118:3462–9. 10.1172/jci34378 18802477 PMC2542847

[57] İscan B Tuzun F Eroglu Filibeli B Cilekar Micili S Ergur BU Duman N Effects of maternal folic acid supplementation on airway remodeling and allergic airway disease development. J Matern Fetal Neonatal Med. (2019) 32:2970–8. 10.1080/14767058.2018.1452904 29587542

[58] Gomes S Lopes C Pinto E. Folate and folic acid in the periconceptional period: recommendations from official health organizations in thirty-six countries worldwide and WHO. Public Health Nutr. (2016) 19:176–89. 10.1017/s1368980015000555 25877429 PMC10270901

[59] Organization WH. Prevention of neural tube defects. Standards for maternal and neonatal care. Geneva: WHO, Department of Making Pregnancy Safer (2006).

