# Supplementary material for: Relationship between maternal folic acid supplementation during pregnancy and risk of childhood asthma: Systematic review and dose-response meta-analysis
Source: Front Pediatr. 2022 Nov 17;10:1000532. doi: 10.3389/fped.2022.1000532 (PMC9714269; doi:10.3389/fped.2022.1000532)
Supplement: Supplementary file 1 [file Table1.docx]

**Annex I:** Search strategy

**PubMed**

| **Search** | **Query** | **Results** |
| --- | --- | --- |
| 1 | "Folic Acid"[Mesh] | 40390 |
| 2 | (((((((Folic Acid[Title/Abstract]) OR (Vitamin M[Title/Abstract])) OR (Vitamin B9[Title/Abstract])) OR (B9, Vitamin[Title/Abstract])) OR (Pteroylglutamic Acid[Title/Abstract])) OR (Folvite[Title/Abstract])) OR (Folacin[Title/Abstract])) OR (Folate[Title/Abstract]) | 45742 |
| 3 | ("Folic Acid"[Mesh]) OR ((((((((Folic Acid[Title/Abstract]) OR (Vitamin M[Title/Abstract])) OR (Vitamin B9[Title/Abstract])) OR (B9, Vitamin[Title/Abstract])) OR (Pteroylglutamic Acid[Title/Abstract])) OR (Folvite[Title/Abstract])) OR (Folacin[Title/Abstract])) OR (Folate[Title/Abstract])) | 61849 |
| 4 | "Asthma"[Mesh] | 136883 |
| 5 | (Asthma[Title/Abstract]) OR (Asthmas[Title/Abstract]) | 162159 |
| 6 | ("Asthma"[Mesh]) OR ((Asthma[Title/Abstract]) OR (Asthmas[Title/Abstract])) | 189152 |
| 7 | (("Folic Acid"[Mesh]) OR ((((((((Folic Acid[Title/Abstract]) OR (Vitamin M[Title/Abstract])) OR (Vitamin B9[Title/Abstract])) OR (B9, Vitamin[Title/Abstract])) OR (Pteroylglutamic Acid[Title/Abstract])) OR (Folvite[Title/Abstract])) OR (Folacin[Title/Abstract])) OR (Folate[Title/Abstract]))) AND (("Asthma"[Mesh]) OR ((Asthma[Title/Abstract]) OR (Asthmas[Title/Abstract]))) | 111 |

**Web of Science**

| **Search** | **Query** | **Results** |
| --- | --- | --- |
| 1 | Folic Acid (TS) or Vitamin M (TS) or Vitamin B9 (TS) or B9, Vitamin (TS) or Pteroylglutamic Acid (TS) or Folvite (TS) or Folacin (TS) or Folate (TS) | 71205 |
| 2 | Asthma (TS) or Asthmas (TS) | 203801 |
| 3 | #1 AND #2 | 264 |

**Embase**

| **Search** | **Query** | **Results** |
| --- | --- | --- |
| 1 | 'folic acid'/exp | 69753 |
| 2 | 'folic acid':ab,ti OR 'vitamin m':ab,ti OR 'vitamin b9':ab,ti OR 'b9, vitamin':ab,ti OR 'pteroylglutamic acid':ab,ti OR folvite:ab,ti OR folacin:ab,ti OR folate:ab,ti | 58974 |
| 3 | #1 OR #2 | 85718 |
| 4 | 'asthma'/exp | 294998 |
| 5 | asthma:ab,ti OR asthmas:ab,ti | 238910 |
| 6 | #4 OR #5 | 327196 |
| 7 | #3 AND #6 | 537 |

**Cochrane Library**

| **Search** | **Query** | **Results** |
| --- | --- | --- |
| 1 | MeSH descriptor: [Folic Acid] explode all trees | 3728 |
| 2 | (Folic Acid):ti,ab,kw OR (Vitamin M):ti,ab,kw OR (Vitamin B9):ti,ab,kw OR (B9, Vitamin):ti,ab,kw OR (Pteroylglutamic Acid):ti,ab,kw | 8506 |
| 3 | (Folvite):ti,ab,kw OR (Folacin):ti,ab,kw OR (Folate):ti,ab,kw | 2444 |
| 4 | #1 or #2 or #3 | 11061 |
| 5 | MeSH descriptor: [Asthma] explode all trees | 12173 |
| 6 | (Asthma):ti,ab,kw OR (Asthmas):ti,ab,kw | 33625 |
| 7 | #5 or #6 | 33632 |
| 8 | #4 and #7 | 87 |
